# Simultaneous Molecular Detection of *Salmonella enterica* Serovars Typhi, Enteritidis, Infantis, and Typhimurium

**Published:** 2017-01

**Authors:** Reza RANJBAR, Seyyed Mojtaba MORTAZAVI, Ali MEHRABI TAVANA, Meysam SARSHAR, Ali NAJAFI, Rahim SORURI ZANJANI

**Affiliations:** 1. Molecular Biology Research Center, Baqiyatallah University of Medical Sciences, Tehran, Iran; 2. Dept. of Microbiology, Faculty of Medicine, Baqiyatallah University of Medical Sciences, Tehran, Iran; 3. Health Management Research Center, Baqiyatallah University of Medical Sciences, Tehran, Iran; 4. Dept. of Public Health and Infectious Diseases, Sapienza University of Rome, Rome, Italy

**Keywords:** *Salmonella* spp, Rapid detection, Multiplex PCR

## Abstract

**Background:**

*Salmonella enterica* serovar Typhi, as causative agent of typhoid fever, is one of the most important endemic pathogens. Non-typhoidal *Salmonella* serovars, including Typhimurium, Infantis, and Enteritidis are amongst the most prevalent serotypes worldwide and in developing areas such as Iran. The aim of this study was to apply a uniplex PCR for rapid detection of *Salmonella* spp., and a multiplex PCR for the simultaneous detection of the four most common *Salmonella* serovars in Iran.

**Methods:**

Current research was done in 2010 at Molecular Biology Research Center, Baqiyatallah University of Medical Sciences, Tehran, Iran. For detection of *Salmonella* spp a pair of primers was used to replicate a chromosomal sequence. Four other sets of primers were also designed to amplify the target genes of four *Salmonella* species including *S. typhi*, and three non-typhoidal *Salmonella* spp (*S. enteritidis, S. infantis, and S. typhimurium*). The assay specificity was investigated by testing 15 different *Salmonella* serovars and 8 other additional non-*Salmonella* species.

**Results:**

The *Salmonella* genus-specific PCR yielded the expected DNA band of 404 bp in all *Salmonella* spp., strains tested. The uniplex and multiplex PCR assays produced also the expected fragments of 489 bp, 304 bp, 224 bp, and 104 bp for serovars Typhi, Enteritidis, Typhimurium, and Infantis, respectively. Each species-specific primer pair set did not show any cross-reactivity when tested on other *Salmonella* serovars or other non- but related- *Salmonella* strains.

**Conclusion:**

Both uniplex and multiplex PCR protocols had a good specificity. They can provide an important tool for the rapid and simultaneous detection and differentiation of the four most prevalent *Salmonella* serovars in Iran.

## Introduction

*Salmonella* is considered as one of the most important causes of acute gastroenteritis and food-borne infections worldwide (1). Gastroenteritis and diarrheal diseases remain one of the most important health problems worldwide, especially in developing countries (2–3). *Salmonella* species are prevalent throughout of the world (4) including Iran (5). This organism can cause a range of clinical outcomes, from an auto-limited gastroenteritis to a systemic infection that compromises the patient’s life (6). The genus *Salmonella* belongs to the family Enterobacteriaceae and consists of two species, *S. enterica* and *S. bongori* (7). *S. enterica* is divided into the following six subspecies: *S. enterica* subsp. *enterica* (I), *S. enterica* subsp. *salamae* (II), *S. enterica* subsp. *arizonae* (IIIa), *S. enterica* subsp. *diarizonae* (IIIb), *S. enterica* subsp. *houtenae* (IV) and *S. enterica* subsp. *ndica* (VI) (8).

At present, over 2600 serovars have been identified, but 99% of the *Salmonella* strains isolated from humans and warm-blooded animals usually belong to group I (*S. enterica* subsp. *enterica*) (9).

Systemic infection caused by *Salmonella* is usually host-dependent (10). Young peoples who have a weakened immune system and animals under the stress are more vulnerable to systemic *Salmonella* infections (11). *Salmonella enterica* servar Typhi (*S. typhi*) causes only systemic infection –typhoid fever–in humans (10, 12).

Typhoid fever as an endemic infectious disease in many developing countries is most important health problems (13) and sometimes is imported in developed countries from travelers or migrants (14, 15). Infections caused by *Salmonella* spp. are increasing in Iran (16).

The US Centers for Disease Control and Prevention (CDC) reported that Nontyphoidal *Salmonellae* were responsible for around 1.4 million cases of gastrointestinal disease with the mortality rates of 500 deaths each year in United States (17). Among the non-typhoidal *Salmonella* serovars, Typhimurium, Infantis, and Enteritidis are amongst the most prevalent serotypes worldwide and in developing areas such as Iran, Southeast Asia and Africa (18–24).

Culture-based microbiological procedures and biochemical/ serological tests are most commonly used methods for detection and identification of *Salmonella* spp. however, these methods are time-consuming resulting in delay in diagnosis, treatment and control of infections. Hence, there is a need to develop “quick and cheap” molecular methods for a rapid and simultaneous detection of the most common *Salmonella* serovars (16, 25, 26). In recent years, some new molecular methods are introduced for rapid detection of *Salmonella* particularly those techniques with good reproducibility and rapidity. PCR and similar nucleotide-based methods have become potentially powerful alternative approaches in microbiological diagnostics because of their higher user-friendliness, rapidity, reproducibility, accuracy and affordability (12, 16, 26–28).

Multiplex PCR is considered as a rapid molecular approach for simultaneous detection of several targets a single amplification reaction. This technique is frequently evaluated in order to assess the possible presence of microbial pathogens causing foodborne diseases (12, 25, 29– 34).

The aim of this study was to apply a uniplex PCR for rapid detection of *Salmonella* spp., and a multiplex PCR for the simultaneous detection of the four most common *Salmonella* serovars in Iran.

## Materials and Methods

### Bacterial strains

Twenty-three strains including 15 *Salmonella* spp and 8 non-*Salmonella* spp were used for the test. *Salmonella* isolates included 12 clinical and 3 standard strains belonging to different serovars. Clinical *Salmonella* isolates were recovered from patients with *Salmonella* infections admitted to including Children’s Medical Center and Baqiyatallah Hospital in Tehran, Iran, during 2008–2010.

As shown in Table 1, the bacterial positive controls used in this study were *S. typhi* (ATCC 19430), *S. typhimurium* (ATCC 14028), *S. infantis* (clinical strains) and *S. enteritidis* (ATCC 4931). Additional bacterial pathogens including *Campylobacter jejuni* (ATCC 33560), *Escherichia coli* (ATCC 25922), *Enterococcus faecalis* (PTCC 1393), *Klebsiella oxytoca* (ATCC 68831), *Shigella sonnei* (ATCC 9290), *Vibrio cholerae* (PTCC 1611), *Proteus mirabilis* (PTCC 1076), and *Serratia marcescens* (ATCC 14223) were used to check the specificity of the assay.

**Table 1:** *Salmonella* species and serovars and non-*Salmonella* micro organisms included in this study

**Strains**	**Reference**	***Salmonella* spp-specific PCR results**	***S.* Typhimurium-specific PCR results**	***S.* Typhi - specific PCR results**	***S.* Enteritidis - specific PCR results**	***S.* Infantis-specific PCR results**
*Salmonella* spp.						
*S.* Albany	Clinical strain	+	−	−	−	−
*S.* Enteritidis	ATCC 4931	+	−	−	+	−
*S.* Hadar	Clinical strain	+	−	−	−	−
*S.* Haifa	Clinical strain	+	−	−	−	−
*S.* Havana	Clinical strain	+	−	−	−	−
*S.* Infantis	Clinical strain	+	−	−	−	+
*S.* Kentucky	Clinical strain	+	−	−	−	−
*S.* Mnchen	Clinical strain	+	−	−	−	−
*S.* Newport	Clinical strain	+	−	−	−	−
*S.* Orion	Clinical strain	+	−	−	−	−
*S.* Paratyphi *B*	Clinical strain	+	−	−	−	−
*S.* Reading	Clinical strain	+	−	−	−	−
*S.* Richmond	Clinical strain	+	−	−	−	−
*S.* Typhi	ATCC 19430	+	−	+	−	−
*S.* Typhimurium	ATCC 14028	+	+	−	−	−
Non-*Salmonella* organisms						
*Campylobacter jejuni*	ATCC 33560	−	−	−	−	−
*Enterococcus faecalis*	PTCC 1393	−	−	−	−	−
*Escherichia coli*	ATCC 25922	−	−	−	−	−
*Klebsiella oxytoca*	ATCC 68831	−	−	−	−	−
*Shigella sonnei*	ATCC 9290	−	−	−	−	−
*Vibrio cholerae*	PTCC 1611	−	−	−	−	−
*Proteus mirabilis*	PTCC 1076	−	−	−	−	−
*Serratia marcescens*	ATCC 14223	−	−	−	−	−

Identification of the references and clinical strains was confirmed by culture, biochemical testing by the API test system (BioMérieux, Marcy-l’Étoile, France) and slide agglutination with serovar specific antisera (Staten Serum Institute, Copenhagen, Denmark) (5, 28). All strains were grown on Trypticase Soy Broth (TSB) and incubated at 37 °C for 18 to 24 h to obtain a fresh culture prior to DNA extraction.

### Bacterial DNA extraction

Three ml of an overnight culture of each *Salmonella* isolate in LB broth were centrifuged at 9000 rpm for 10 min.

Genomic DNA of the *Salmonella* strains was extracted using a DNA extraction Kit (Roche, Germany) according to the manufacturer’s instruction. The supernatant containing the DNA was transferred to a clean tube and stored at −20 °C until used for PCR.

### Genomic PCR targets and primers

Five sets of primers were designed to amplify the target genes of *Salmonella* spp and of four *Salmonella* serovars, and the three most prevalent nontyphoidal *Salmonella* serovars in Iran*.* The list of the primers and their sequences are presented in Table 2. To avoid cross-reactivity with *Salmonella* related bacteria and within each other *Salmonella* serovar, genus and species specific regions of the *Salmonella* genome were considered to design the primers, respectively. The target genes included *invA* for *Salmonella* spp., *STY4669* (a hypothetical protein) for Typhi; *STMO159* (a putative restriction endonuclease) for Typhimurium; *SEN1383* (a hypothetical protein) for Enteritidis, and *M. sini* (a modification methylase) for Infantis.

**Table 2:** The primers and their sequences used in the study

**Bacterial strains**	**Target gene**	**Sequence of the primers**	**PCR Size (bp)**
*Salmonella* spp	invA-secretory protein	F: 5′-GTATTGTTGATTAATGAGATCCG-3′ R: 5′-ATATTACGCACGGAAACACGTT-3′	404
*S*. Typhi	STY4669 - hypothetical protein	F: 5′-TGTCCGCTGTCTGAAGTCATC-3′ R: 5′-ATCTCAGGCAAACTCACAAGGG-3′	489
*S.* Typhimurium	STM0159 - restriction endonuclease	F: 5′-ATGATGCCTTTTGCTGCTTT-3′ R: 5′-TCCCAGCTCATCCAAAAA-3′	224
*S*. Enteritidis	SEN1383 - hypothetical protein	F: 5′-TGTGTTTTATCTGATGCAAGAGG-3′ R: 5′-TGAACTACGTTCGTTCTTCTGG-3′	304
*S*. Infantis	M.SinI -modification methylase	F: 5′-CACAATGAACGTGGTGAAGG-3′. R: 5′-CGTCCCGCGAACATATTATT-3′	184

### Uniplex PCR and Multiplex PCR

A uniplex PCR assay was first evaluated using bacterial colonies isolated from pure cultures of clinical strains of each *Salmonella* serovar. The concentration of each primer pair, magnesium chloride concentration, and primer annealing temperature were optimized before checking for the specificity of the primer pairs. The optimized PCR was carried out by using a total volume of 25 μL containing 1× PCR buffer, 1 mM MgCl2, 1 U *Taq* DNA polymerase (Fermentas, Lithuania), 200 μM dNTPs (Fermentas, Lithuania), 0.5 μM of each primers and 2.5 μL of DNA template. The PCR program used for amplification consisted of 5 min at 95 °C, followed by 30 cycles of 60 sec at 95 °C of denaturing temperature, 60 sec at 57 °C of annealing temperature, and 5 min at 72 °C of extension temperature. At the end of the 30 cycles, a 10 min extension at 72 °C was used. Each multiplex PCR mixture in one reaction was prepared by using a total volume of 25 μL containing 0.5 μM of each primer (five pairs), 2,5 μL PCR buffer 10X, 1.5 U Taq DNA polymerase (Fermentas, Lithuania), 1.5 mM MgCl2, 200 μM dNTPs (Fermentas, Lithuania) and 1 μL DNA template. The multiplex PCR was carried out through 30 cycles following a pre-heat step at 95 °C for 5 min. Each cycle consisted of denaturation at 95 °C for 60 sec, annealing at 57 °C for 1min, and extension at 72 °C for 1min. After the 30 cycles, samples were maintained at 72 °C for 10 min. Sterile distilled water was included in each PCR assay as a negative control. The amplified DNA was separated by 1.5% agarose gel electrophoresis, stained with ethidium bromide, and visualized by UV transillumination. To prevent any contamination, reaction mixture preparation, DNA amplification and gel migration were done in separate rooms.

## Results

Both uni and multiplex PCR assays produced the expected fragments bands when applied on standard and clinical strains of *Salmonella*.

**Fig. 1:**
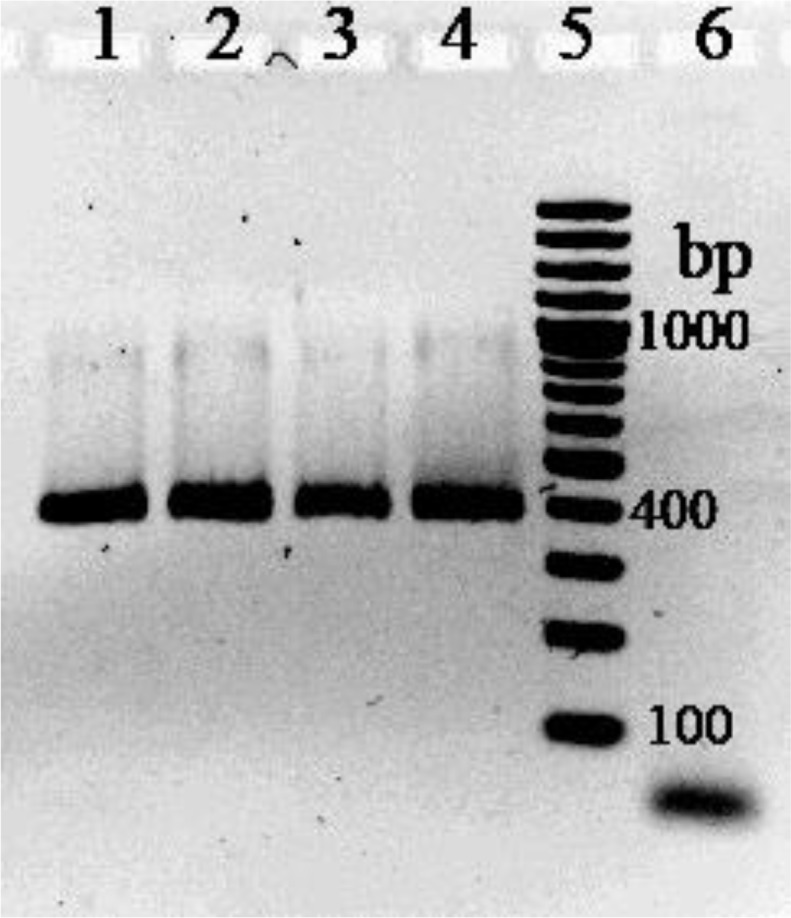
*Salmonella* spp specific PCR. Lanes 1–4: PCR products from some representative strains of *S.* Infantis, *S.* Enteritidis, *S.* Typhimurium and *S.* Typhi, respectively. Lane 5: molecular weight (100bp DNA ladder). Lane 6: *E. coli*

**Fig. 2:**
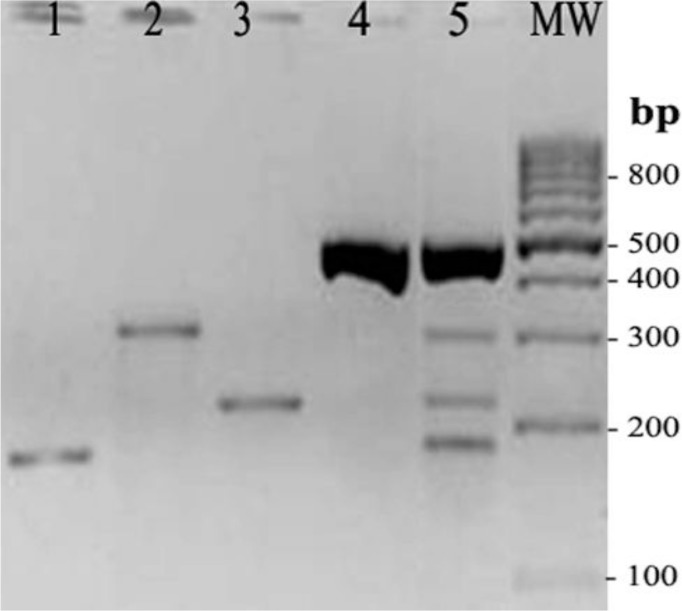
Multiplex PCR. Lanes 1–4: uniplex PCR of some representative strains of *S.* Infantis, *S*. Enteritidis, *S*. Typhimurium and *S*. Typhi, respectively. Lane 5: multiplex PCR for the same four *Salmonella* serovars in a single PCR tube. MW: molecular weight (100 bp DNA ladder)

Uniplex PCR targeting for *Salmonella* genus specific gene showed the expected amplified DNA band in all *Salmonella* spp. strains tested and any non-specific reaction with other non *Salmonella* spp., strains was seen. Fig. 1 shows the specific band of 404 bp obtained from some *Salmonella* serovars tested. Multiplex PCR assays produced the expected fragments of 489 bp, 304 bp, 224 bp, and 104 bp for serovars Typhi, Enteritidis, Typhimurium and Infantis, respectively. No amplification products were observed with any of the other *Salmonella* serovars as well as with non *Salmonella* strains indicating the good specificity of the test. Fig. 2 shows the specifically amplified bands obtained by multiplex PCR on the four *Salmonella* serovars.

## Discussion

The invasion protein A gene is a good target for rapid and specific detection of *Salmonella* spp. This protein plays a part in cytoskeletal rearrangements for internalization of *Salmonella* spp. (35).

In a previous study, PCR using the invA primers has shown to be specific for detection of *Salmonella* spp (36). Our result confirmed the finding of previous reports, which indicated, this gene is present in nearly all *Salmonella* spp. (34, 35).

We also designed a new multiplex PCR with four sets of primers to identify Typhi and the most common non-typhoidal *Salmonella* serovars in our country. Accordingly, the designed method was able to detect all the *Salmonella* strains under study. Any non-specific reaction with other non *S. enterica* strains was not observed confirming that, this assay is specific for detection of *Salmonella* spp.

Several molecular techniques such as Multiplex PCR were evaluated for detection of foodborne pathogen organisms including *Salmonella* spp. However, among them; Multiplex PCR is considered as a rapid approach for simultaneous detection and identification of several targets in a mix-contaminated sample (12, 15, 25, 29–32). Optimization of reaction parameters and, in particular, of annealing temperature is very critical in this technique. The annealing temperature of 57 °C proved to be optimal for detection and differentiation of the four *Salmonella* serovars under study. The assessment of multiplex PCR analysis using standard and clinical samples showed that this method PCR was reliable for simultaneous detection of *Salmonella* strains belonging to different species. PCR results obtained from the clinical samples were consistent with results from the standard strains.

Some researchers have introduced some multiplex PCR for rapid detection and differentiation of endemic and prevalent *Salmonella* serovars in their countries (6,12,14,15,18).

*Salmonella typhi* and *paratyphi* A could be detected and differentiated using five sets of primer pairs targeting against the *viaB*, *prt*, *tyv*, *fliC-d*, and *fliC-a* genes (33). The conventional multiplex PCR evaluated to detect the causative agents of typhoid and paratyphoid fever based on *tyv* (*rfbE*), *prt* (*rfbS*) and *invA* genes. The multiplex PCR was indicated a potentially valuable tool for rapid diagnosis of typhoid *Salmonella* (15).

Multiplex PCR was a simple, inexpensive and sensitive molecular method for the rapid detection of the most prevalent *Salmonella* serovars in human clinical samples. They used six different primers for amplification of some target genes in *Salmonella* spp., DT104 and U302, S. C2 serogroup and *Salmonella* serovar 4,5,12:i:- and found their multiplex PCR assay can consider as an appropriate alternative approach for subtyping of *Salmonella* serovars (27).

*Salmonella* isolates identified in chicken abattoirs from Sao Paulo State, Brazil by a multiplex-PCR using three sets of primers targeting the *invA*, *pefA*, and *sefA* gene sequences from *Salmonella* spp., Typhimurium and Enteritidis, respectively (31).

The multiplex PCR designed to detect and identify *Salmonella* spp,. Enteritidis and Typhimurium strain isolated from fecal samples of diseased cattle, sheep and chicken in Egypt. They selected three PCR target sequences, which were *invA* specific for the genus *Salmonella*, *spvA* specific for Enteritidis and *fliC* gene specific for Typhimurium. Their results showed that Typhimurium and Enteritidis could be distinguished in less than 4 h (34).

A multiplex PCR introduced to detect *Salmonella* genus and serovars in refrigerated carcasses and chicken viscera and showed that the multiplex PCR was able to detect the presence of these serovars in a short period of time (25).

A multiplex PCR assay established to identify the seven major serovars of *Salmonella*, i.e., Typhimurium, Choleraesuis, Infantis, Hadar, Enteritidis, Dublin and Gallinarum circulating in Japan. Multiplex PCR had sufficient simplicity and specificity to identify the seven *Salmonella* serovars, and therefore, had the potential for use as rapid screening methods (12).

Ngan et al developed an mPCR assay for specific detection of serovars Typhi and Paratyphi A. They evaluated their assays with 124 clinical and reference strains of *Salmonella* serovars and found that *S. enterica* serovars Typhi and Paratyphi A can be detected with high specificity and sensitivity. They showed that mPCR could prove to be a useful diagnostic tool for the detection and differentiation of serovars Typhi and Paratyphi A (37).

Recently, an mPCR was described for identification of *Salmonella* spp. They could differentiate simultaneously different serovars of *S. enterica* (38).

We tested our method on DNA extracted from pure bacterial colonies. It is the major limitation of the study. To achieve a powerful assay for rapid detection and differentiation of *Salmonella* serovars, it is better to evaluate our method directly on clinical samples in the future.

The second limitation was to apply the test for rapid detection of other *Salmonella* serovars, however, they are not prevalent in our country. Moreover, optimization of multiplex PCR would be more difficult for simultaneous detection of more than 4 different bacterial species.

The choice of a multiplex PCR method, will depend on the type of microorganisms particularly those are most prevalent and the aim and scope of the study.

With the new more sophisticated molecular technologies, a new era seems to be opening in the field of diagnostic and molecular epidemiology of infectious diseases in which a single method will likely apply for detection of different targets (39).

## Conclusion

The technique presented here showed to be specific for detection of *Salmonella* spp. and differentiation of the four *Salmonella* serovars tested. No false positive results occurred during the assay indicating that target genes used in the study were specific for these *Salmonella* serovars. The multiplex PCR using four primers sets was able to detect four serovars of *Salmonella* simultaneously in a single reaction by the combinations of the different size amplicons without any cross-reactivity. The method presented here showed a good specificity and proved to be able to provide an important diagnostic tool for the rapid and simultaneous detection of the four most prevalent serovars of *Salmonella* in Iran.

## Ethical considerations

Ethical issues (Including plagiarism, informed consent, misconduct, data fabrication and/or falsification, double publication and/or submission, redundancy, etc.) have been completely observed by the authors.
